# Caregiver burden and its associated factors among family caregivers of persons with dementia in Athens, Greece: a cross sectional study

**DOI:** 10.1192/j.eurpsy.2023.2090

**Published:** 2023-07-19

**Authors:** K. Argyropoulos, P. Antzoulatos, A. Argyropoulou, D. Avramidis, P. Gourzis, E. Jelastopulu

**Affiliations:** 1 Postgraduate Program “Aging and Chronic Diseases Management”, School of Medicine, University of Thessaly & Hellenic Open University, Greece, Athens; 2 School of Medicine, University of Patras, Greece; 3 School of Medicine, Univesrity of Patras, Greece; 4 Psychiatry; 5 Public Health, School of Medicine, University of Patras, Patras, Greece

## Abstract

**Introduction:**

Studies have shown that dementia family caregivers to be significantly more burdened than non-dementia caregivers.

**Objectives:**

The aim of the present study was to analyze factors affecting the quality of life and the burden of dementia family caregivers.

**Methods:**

70 dementia family caregivers who lived in the Attica Region, Greece participated in the study from February to April 2022. An anonymous questionnaire was used including 16 items regarding demographic and socio-economic factors. The 22 -item Zarit Burden scale was used to estimate the burden of dementia family caregivers. Statistical analysis was performed with SPSS 21.

**Results:**

1.4% of caregivers showed minimal to no burden (n = 1). 28% of caregivers (n = 20) a mild to moderate burden. 40.6% (n = 29) presented a moderate to severe burden, while 28% (n = 20) a very serious burden. According to the results of the present study, there are three main factors that affect the quality of life of caregivers. Caregivers who spend more time with the patient have an increased burden compared to caregivers who spend less time. The patient’s low Mini Mental score is associated with an increase in burden. Caregivers who have attended training and management programs for the care of a patient with dementia have a lower burden than those who have not attended programs.

**Conclusions:**

The study highlights an increased burden on caregivers. Social supports with multiple coping strategies focusing on different levels of patients with dementia and caregivers’ needs should be planned to relieve the caregiver burden.

**Disclosure of Interest:**

None Declared

